# Tuning Hydrogel Mechanics and Microstructure to Maximize Extracellular Vesicle Production from Mesenchymal Stem Cells

**DOI:** 10.1007/s12195-026-00917-x

**Published:** 2026-06-03

**Authors:** Riddhesh B. Doshi, Bethany Yee, Nicolas Warburton, Juanfang Ruan, Richard Tilley, Kuldip Sidhu, Kristopher A. Kilian

**Affiliations:** 1 Australian Centre for NanoMedicine, School of Chemistry, University of New South Wales (UNSW), Sydney, NSW 2052 Australia; 2 CK Cell Technologies Pty Ltd, Sydney, NSW 2153 Australia; 3 Electron Microscope Unit, Mark Wainwright Analytical Centre, University of New South Wales (UNSW Sydney), Sydney, NSW 2052 Australia; 4 School of Materials Science and Engineering, University of New South Wales (UNSW), Sydney, NSW 2052 Australia

**Keywords:** Mesenchymal stem cell, Biomaterials, Extracellular vesicles, Microcarriers

## Abstract

**Background:**

The secretory output from mesenchymal stem cells (MSCs) have emerged as promising therapeutics with extracellular vesicles (EVs) gaining prominence due to solution stability and optimal size for overcoming biological barriers during delivery. However, reproducible and scalable production of EVs for therapeutic use remains a challenge in biotechnology. Here we demonstrate optimization of EV production from MSCs using soft hydrogel microcarriers.

**Methods:**

Gelatin methacryloyl (GelMA) hydrogels were prepared at a range of concentrations for the culture of two sources of MSCs: adipose derived stem cells (ADSCs) and induced pluripotent stem cell derived MSCs (iMSCs). The mechanical properties of the hydrogels were evaluated using shear rheology. EVs were isolated and analyzed for physical and biological characteristics using electron microscopy, nanoparticle tracking, proteomics, and functional assays for wound healing and angiogenesis.

**Results:**

Both cell types were responsive to hydrogel stiffness (0.3-16 KPa), showing optimal EV secretion from cultures on 10 KPa hydrogels, with a further 18-fold increase when formulated as microcarriers compared to traditional monolayer culture. Proteomics analysis and functional assays revealed that EVs from microcarrier culture displayed increased wound healing and regenerative properties.

**Conclusion:**

This study demonstrates the advantages of hydrogel microcarriers in the production of cell-derived products, with optimized design parameters to guide scaleup and translation to manufacturing, in support of biotechnology and biomedical applications.

**Supplementary Information:**

The online version contains supplementary material available at 10.1007/s12195-026-00917-x.

## Introduction

Mesenchymal stem cells (MSCs) and their secreted products have proved a compelling option for regenerative medicine and tissue engineering due their immunomodulatory [1], angiogenic [2] and tissue repairing capabilities [3]. These multipotent cells can either be tissue derived: adipose, bone marrow, umbilical cord [4–6] or differentiated from pluripotent stem cells, e.g., iPSC-MSC or ESC-MSC [7, 8] with autologous-patient specific and allogenic-donor specific options. Each category presents distinct advantages and limitations, particularly in terms of scalability, cost, and accessibility. Autologous cells offer personalized treatment with minimal immune rejection but are often more expensive and less scalable. In contrast, allogenic cells enable mass production off the self and broader accessibility, though they may pose immunogenic risks and require more stringent regulatory oversight [9, 10]. These therapeutic cells exert their effects primarily through paracrine signalling rather than direct differentiation. MSCs secrete a diverse array of bioactive molecules, including cytokines, chemokines, nucleic acids and growth factors, that help modulate immune responses and promote tissue regeneration. Recent advancements in MSC therapies have led to a shift toward cell-free alternatives that utilize secreted products, particularly extracellular vesicles (EVs) such as exosomes and microvesicles [11]. These nanoscale vesicles are enriched with proteins, lipids, and nucleic acids that can influence the behavior of recipient cells. This transition to EV-based approaches supports the development of cell-free therapeutic strategies, which offer notable advantages over traditional cell-based treatments including improved safety profiles, greater scalability, and simplified regulatory pathways [12]. As a result, EVs are increasingly recognized as a powerful and versatile tool in regenerative medicine and immunomodulation.

Extracellular vesicles have been actively investigated for wide range of therapeutics applications namely, acute and chronic wound healing, musculoskeletal and neurological disorders, immune diseases, and degenerative conditions, etc. due to their capability to cross biological barriers and present therapeutically relevant molecular signatures [13]. To translate EVs into clinical practice, efficient and scalable manufacturing of EVs requires systems like bioreactors [14, 15] and advanced material platforms like microcarriers [16, 17], scaffolds, and microfluidics [18], for enhanced cell culture environments that will result in commercially/clinically viable yields. Scalable isolation techniques like size exclusion chromatography and tangential flow filtration optimise EV recovery and improve functionality [19, 20]. Additionally, cell conditioning techniques such as hypoxia, chemical stimulation and mechanical stress can significantly boost EV production and tailor their cargo for specific therapeutic outcomes [21–23]. Together, these integrated approaches form the foundation for advancing EVs from bench to bedside, making them a promising frontier in biomedical innovation.

Research has shown that culture substrate stiffness, topography and dimensionality directly affect MSC behaviour and EV secretion. These cues modulate cytoskeletal tension, signalling pathways and EV biogenesis pathways. Lenzini et al. demonstrated that MSCs seeded on soft substrates secrete fivefold more EVs as compared to rigid substrates [24] while Romanazzo et al previously demonstrated that iMSCs seeded on varying ECM coated hydrogel substrate demonstrate higher angiogenic potential [8]. Cell culture substrates that mimic properties of native tissue can boost EV release by activating mechanotransduction through elevated activities of molecules like YAP/TAZ, RhoA and ROCK. Mechanical cues influence cytoskeletal tension and intracellular signalling, directly impacting EV biogenesis and cargo loading. Deformable carriers and 3D scaffolds provide dynamic, physiologically relevant niches that further improve EV consistency and scalability as it closely resembles in vivo tissue architecture as compared to the widely used planar culture substrates. Bioreactor systems using soft microcarriers have reported a multifold increase in EV production compared to traditional 2D cultures. The integration of tunable biomaterials and mechanically active culture systems is therefore a promising strategy for optimizing MSC-EV production for therapeutic applications, especially in regenerative medicine and immunomodulation.

In this study, we demonstrate that by precisely tuning the mechanical properties and microstructure of hydrogels, we can significantly enhance EV production from mesenchymal stem cells. Using a combination of rheological analysis, imaging techniques, and EV quantification assays, we show that specific hydrogel formulations create an optimal niche for vesicle secretion. Our findings suggest that material design is not merely a passive scaffold strategy but a powerful tool for actively directing cell function. This work lays the foundation for next-generation EV manufacturing platforms that integrates biomaterials science with cellular engineering.

## Materials and Methods

### Synthesis of Gelatin Methacryloyl (GelMA)

GelMA was synthesized following established protocols [25]. Type A gelatin (porcine skin, Bloom strength 300; Cat: G2500 Sigma-Aldrich) was dissolved at 10% w/v in phosphate-buffered saline (PBS, pH 7.4) at 50 °C under continuous stirring until fully solubilized. Methacrylic anhydride was then added at 5% v/v relative to the total volume, and the reaction mixture was stirred for 90 minutes while maintaining the temperature at 50 °C. Following this, the solution was diluted two-fold with PBS and centrifuged at 3000 rcf for 3 minutes to remove unreacted methacrylic anhydride. The resulting supernatant was subjected to dialysis using a 14 kDa molecular weight cutoff membrane (Sigma-Alrich, D9777) at 40 °C for 5 days, with daily replacement of the dialysis water. Finally, the purified GelMA was lyophilized for 5 days and stored at − 20 °C for long-term use.

### Preparation and Coating of Glass Coverslips with GelMA Hydrogel

18 mm round glass coverslips were sequentially sonicated in MilliQ water and ethanol for 15 minutes each. After rinsing with ethanol, the coverslips were incubated at room temperature for 2 hours in a solution containing 2% TMSPMA, 6% glacial acetic acid, and 92% ethanol to introduce methacrylate functional groups. Post-functionalization, coverslips were washed with ethanol and dried using nitrogen gas.

GelMA was dissolved in 1 × PBS overnight at varying concentrations, 100 µl of GelMA solution was then mixed with 2 µl of 2.5% (w/v) LAP (Sigma-Aldrich, 900889) photo-initiator solution. A 20 µL aliquot of the mixture was placed between a Rain-X treated hydrophobic glass slide and the TMSPMA-treated coverslip. The assembly was exposed to 395 nm UV light torch for 2 minutes to initiate crosslinking. After gelation, the coverslips were carefully separated from the glass slide and washed three times with PBS. The GelMA-coated coverslips were sterilized under UV light in a biosafety cabinet and rinsed with PBS prior to cell seeding.

### GelMA Microcarrier Synthesis

GelMA microcarriers were synthesized using a modified water-in-oil emulsion technique. Lyophilized GelMA was rehydrated to a 10% (w/v) solution in 1 × PBS at 40 °C [26]. This solution was filtered through a 0.45 μm membrane filter and introduced dropwise into a continuously stirred oil bath (canola, sunflower, or olive oil) maintained at 40 °C. Emulsification was allowed to proceed for 10 minutes. The temperature was then reduced to 10 °C for 20 minutes, followed by the addition of acetone at a ratio of 22 mL per mL of GelMA solution to dehydrate the microcarriers. The microcarriers were allowed to settle, washed thoroughly with acetone, and sonicated for 10 seconds to disperse aggregates. Unhydrated aggregates were removed via filtration. The dehydrated microcarriers were stored in acetone until further use.

For hydrating microcarriers, acetone was removed by evaporation. For 100 mg of dried microcarriers, 1.8 mL of Low Glucose (LG) DMEM and 0.037 mL of 2.5% (w/v) LAP was added [26]. A volume of 100 μL of the hydrated microcarrier suspension was dispensed per well and photo-crosslinked using a 395 nm UV light torch for 2 minutes.

### Rheology

All rheological measurements were performed on an Anton Paar MCR 302 rheometer with a parallel plate geometry (25-mm disk, 1-mm measuring distance, 600 μL of GelMA solution). Oscillatory measurements were performed with 0.02% strain and a 1 Hz of frequency for the duration of gelation at 37 °C for 15 minutes. For in situ UV cross-linking of the GelMA hydrogels, a UV light (with 405 nm UV light at 40 mW/cm^2^ for 120 s) was placed underneath to illuminate the sample through the quartz crystal stage.

### Cell Culture

Induced pluripotent stem cell-derived mesenchymal stem cells (iMSCs) were obtained from CK Cell Technologies Pty. Ltd., and human adipose-derived stem cells (hADSCs) were purchased from ATCC. Both cell types were maintained in low-glucose Dulbecco’s Modified Eagle Medium (LG DMEM) supplemented with 10% Fetal bovine serum (FBS), 1% penicillin/streptomycin, and 5 ng/mL fibroblast growth factor-2 (FGF-2), collectively referred to as MSC expansion medium. Cells were utilized between passages 4 and 9. For the collection of conditioned media, LG DMEM devoid of FBS and antibiotics was used.

Human Fetal fibroblasts (hFFs) were cultured in LG DMEM supplemented with 15% FBS and 1% penicillin/streptomycin and utilized between passages 16 and 20. Human microvascular endothelial cells (HMVECs; Lonza) were expanded using the EGM-2MV Bullet Kit (components CC-3156 and CC-4147, Lonza) and used between passages 4 and 9.

### Isolation of Extracellular Vesicles from MSC-Conditioned Media

Following aspiration of MSC expansion medium, cells were rinsed twice with PBS to remove residual media and subsequently cultured in low-glucose DMEM for 24 hours to generate MSC-conditioned media (MSC-CM). After conditioning, MSC-CM was collected and centrifuged at 3500 × g for 5 minutes to eliminate cellular debris. The supernatant was then filtered through a 0.45 μm sterile syringe filter.

Extracellular vesicles were isolated using the Total Exosome Isolation Reagent (Thermo Fisher Scientific, Cat. No. 4478359) according to the manufacturer’s instructions. Briefly, MSC-CM was mixed with the reagent at a 2:1 ratio (CM: reagent) and incubated overnight at 4 °C. The mixture was centrifuged at 10,000 × g for 1 hour at 4 °C, and the resulting supernatant was discarded. The EV pellet was resuspended in 100 μL of 4% trehalose in PBS and stored at − 80 °C until further use or lyophilized.

### Extracellular Vesicle Quantification

Isolated EV samples were thawed immediately prior to analysis, vortexed briefly, and diluted with sterile 1 × PBS. Quantification was performed using the NanoSight NS300 system at 25 °C. For each sample category, three 30-second measurements were recorded to ensure consistency and accuracy.

### Cell Seeding

For 2D cell seeding, MSCs were passaged using 0.05% Trypsin-EDTA, counted, centrifuged, and resuspended at a density of 10^5^ cells/ml in MSC expansion medium. A 1 ml aliquot of the cell suspension was added to each gel-coated coverslip.

For GelMA bulk encapsulation, MSCs were resuspended at 10^6^ cells/ml in GelMA solution containing LAP. A 0.1 ml volume of the cell–GelMA suspension was dispensed into each well, UV crosslinked for 2 minutes and subsequently overlaid with MSC expansion medium. For GelMA microcarrier (MC) encapsulation, MSCs were resuspended at 10^6^ cells/ml in hydrated GelMA microcarrier solution containing LAP. A 0.1 ml aliquot of the cell-GelMA MC suspension was added to each well, UV crosslinked for 2 minutes and then supplemented with MSC expansion medium.

### Immunostaining and Immunofluorescence

2D GelMA hydrogels were fixed in 4% PFA for 10 mins followed by 3x PBS washes. Next, they were permeabilized for 15 mins in 0.5% Triton X-100 followed by blocking with 1% BSA solution.

GelMA bulk and GelMA microcarrier suspensions were fixed using a 4 wt% paraformaldehyde (PFA) (Chem-Supply) at room temperature overnight to ensure fully penetration of PFA into thick constructs. The gels were then rinsed with PBS followed by 3x PBS washes at 2-4 h intervals (standard washing procedure). A 0.5 wt% solution of Triton X-100 in DI water was added to the gels and allowed to sit at RT overnight. The gels were then rinsed with PBS followed by 3 washes with 3 wt% Bovine Serum Albumin (BSA) in PBS for blocking (2 h wash intervals). DAPI (1:1000) and Phalloidin 488 (1:200) was added to cells on a rocker overnight at room temperature. The gels were washed with PBS three final times before the addition of the CUBIC-2 clearing solution for 2 days. Clearing solutions were prepared as done previously with slight modifications [27]. Briefly, CUBIC-2 solution was prepared by mixing 50 wt% sucrose (Sigma-Aldrich,584173), 25 wt% urea, 10 wt% triethanolamine (Sigma-Aldrich, 90278-100 mL) with DI water at 55 °C until also fully dissolved. All confocal imaging was performed with a Zeiss LSM 800. A 10x objective with a 2.5 mm working distance was used to see deeper into the samples. Samples were coated with CUBIC-2 solutions throughout the duration for the imaging to prevent drying. 2D GelMA hydrogel image analyses were conducted using Fiji ImageJ, while GelMA bulk and GelMA microcarriers images analyses were performed using Imaris software (x64 9.9.1).

### Scratch Assay

HFFs were seeded onto 6-well plate at a seeding density of 2 × 10^5^ cells per well until confluent. Next, a sterile 100-μL pipette tip is used to create a multiple linear scratch, the media was aspirated, each well was washed with PBS to remove unattached cells. ADSC- and iMSC-EVs from TCP and microcarrier conditions were resuspended in LG DMEM and added to each well with LG DMEM negative control. Finally, plates were incubated at 37 °C, 5% CO_2_, and imaged after 24 hrs under an inverted brightfield microscope. Percentage of migrated cell area was calculated as (*A*_0_ − *A*_t_)/*A*_0_ × 100 where *A*_0_ is scratch area at *t* = 0 hrs and *A*_t_ is scratch area at 24 hrs and plotted as % Area covered v/s time. Scratch area was measured using ImageJ software.

### Tube Formation Assay

The in vitro vascularization assay was performed as previously described in a similar study [8]. Briefly, 30 μL of Geltrex LDEV-Free Reduced Growth Factor Basement Membrane Matrix (Thermo Fisher, USA) was used to coat 96-well plates and then incubated at 37 °C for 30 min, to allow gel formation. Subsequently, 1 × 10^4^ HMVECs at passage 4-9 was seeded in each well in 50 μL of Endothelial Basal Media-2 (Lonza, USA) and 100 μL of CM or EV suspension was added to each well. Endothelial Basal Media-2 was used as a negative control, while Endothelial Growth Media-2 was used as a positive control. Tube formation was assessed up to 8 h after cell seeding, by imaging samples using a microscope at 4 × magnification. Tube formation was then quantified using the ImageJ plugin “Angiogenesis analyzer” (written by Gilles Carpentier, 2012, available at:

http://image.bio.methods.free.fr/ImageJ/?Angiogenesis-Analyzer-for-ImageJ#outil_sommaire_0.

### Cryo-EM Data Acquisition

Briefly, 4.5 μL of EV samples were applied to glow discharged Quantifoil R2/2 copper grids (Quantifoil Micro Tools) and blotted for 2.5 seconds in a 95% humidity chamber, then plunged in liquid ethane using a Lecia EM GP device (Leica Microsystem). The grids were imaged using a Talos Arctica Cryo TEM (Thermo Fisher Scientific) and operated at 200 kV, with the specimen maintained at liquid nitrogen temperatures. Images were recorded on a Falcon 3EC direct detector camera operated in linear mode.

### Proteomic Analysis of MSC-Secretome

Extracellular vesicles (EVs) derived from MSCs in each group were collected and subjected to secretome profiling using LC-MS/MS, as previously established by the Bioanalytical Mass Spectrometry Facility at the Mark Wainwright Analytical Centre, UNSW [28]. Peak lists were generated with Mascot Distiller (Matrix Science) and analysed using Mascot (version 2.8.3, Matrix Science). Database searches were performed against UniProt with the following parameters: precursor ion tolerance of 4 ppm, product ion tolerance of ± 0.05 Da, oxidation of methionine (Met(O)) and carboxyamidomethylation of cysteine specified as variable modifications, trypsin as the digestion enzyme, and allowance for one missed cleavage.

Functional enrichment analysis of the identified proteins was conducted using FunRich (version 3.1.3) to determine overrepresented biological processes (BP), cellular components (CC), and molecular functions (MF) based on Gene Ontology (GO) annotations [29].

### Statistical Analysis

All data were presented as mean ± standard deviation (SD) from at least triplicate samples and plotted using GraphPad Prism (GraphPad, San Diego, CA). Student’s *t*-test was used to analyze the data between two groups. For comparing the differences between multiple groups, one-way or two-way analysis of variance was performed. *p < 0 .05, **p < 0 .01, ***p < 0.001 and ****p < 0.0001 was considered statistically significant.

## Results and Discussion

### Gelatin Methacryloyl Content Influences Hydrogel Stiffness and Adherent Cell Morphology

To investigate the influence of substrate mechanics on extracellular vesicle (EV) secretion, adipose-derived mesenchymal stem cells (ADSCs) and iPSC-derived MSCs (iMSCs) were cultured on gelatin methacryloyl (GelMA) hydrogels with varying concentrations. ADSCs were used as a comparative model to evaluate differences in EV secretion between tissue-derived and iPSC-derived MSC lines under mechanically distinct microenvironments. GelMA hydrogels were used due to their ease of use and widescale popularity for 3D cell culture, where photo-mediated crosslinking can precisely tune substrate stiffness [30]. GelMA contains RGD and other adhesion motifs, which facilitates cell engagement of the material through α_v_β_3_ and α_5_β_1_ integrins, which are highly expressed in MSCs.

**Fig. 1:**
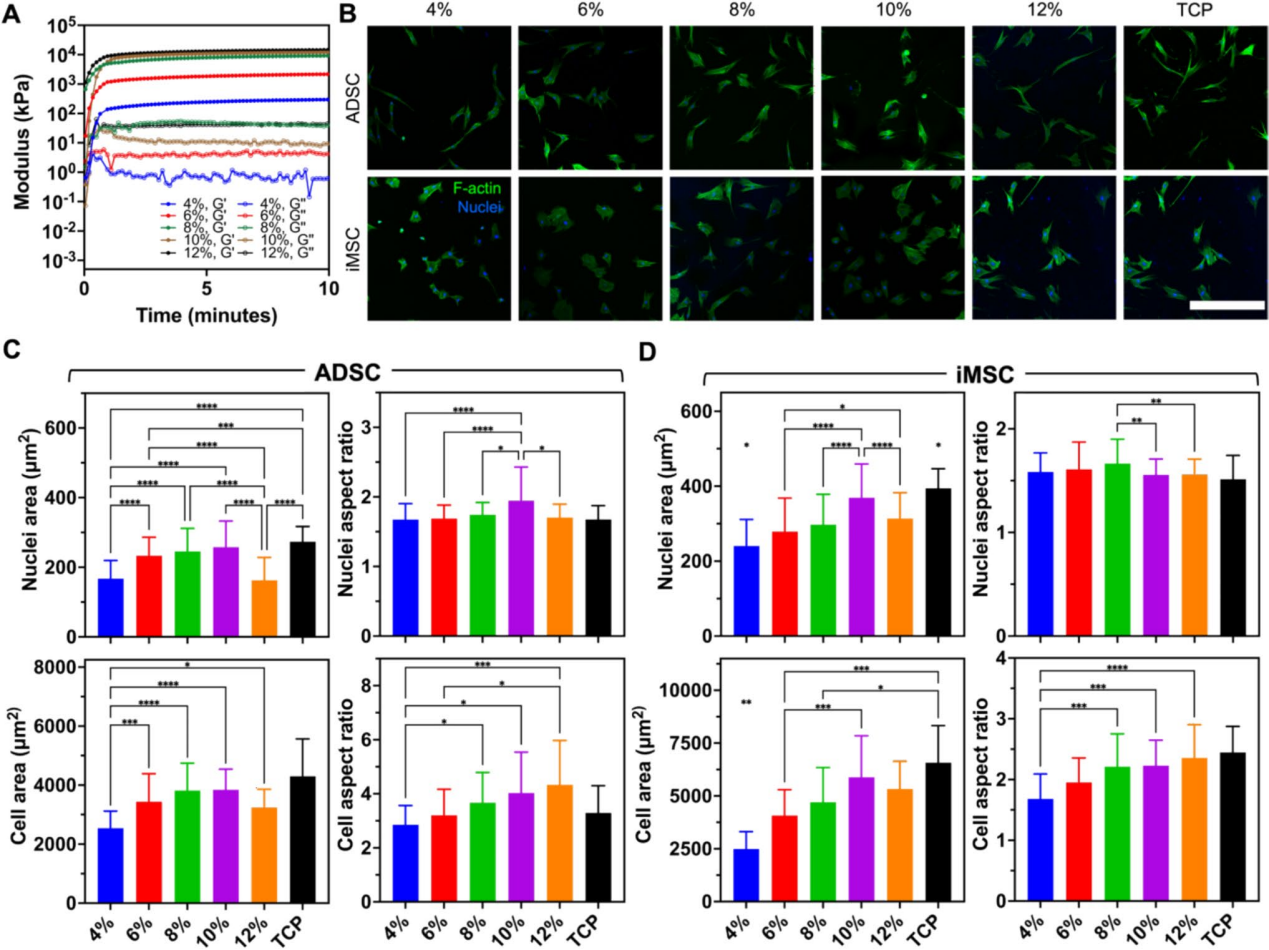
Hydrogel stiffness influences cell adhesive morphology. **A**. increased weight content leads to increased stiffness in GelMA hydrogels. **B** and **C**. ADSCs and iMSCs show differences in cell and nuclear morphometrics after 2 days in culture, Scale bar = 500 µm. Statistical significance determined with *p < 0 .05, **p < 0 .01, ***p < 0.001and ****p < 0.0001

We prepared GelMA hydrogels at different concentrations, which yielded materials with a range of storage modulus from 0.35 (4%), 2.47 (6%), 9.07 (8%), 12.3 (10%) and 15.73 (12%) KPa (Fig. 1A). This range was selected because it corresponds to physiological substrate stiffness necessary for modulating MSC to medicinal phenotype [31]. Immunofluorescence staining of actin and nuclei revealed that both ADSCs and iMSCs undergo notable morphological changes in response to increasing GelMA concentrations (Fig. 1B). From 4 to 10% GelMA, both cell and nuclear areas expanded, suggesting that moderate substrate stiffness enhances cell spreading and nuclear expansion, key indicators of mechano-sensing and cytoskeletal engagement [32]. However, at 12% GelMA, a decline in area was observed, indicating that excessive stiffness may exceed the optimal mechanical threshold for MSCs, potentially inducing a stress-responsive phenotype (Fig. 1C–D).

Additionally, the cell aspect ratio increased with stiffness, reflecting elongation and polarization commonly linked to migratory or activated cell states. In contrast, nuclear aspect ratio changes were modest (Fig. 1C–D). These findings align with prior studies showing that substrate stiffness can modulate MSC behaviour and differentiation through mechanotransduction pathways such as YAP/TAZ and Rho/ROCK signaling [32].

### Hydrogel Stiffness Directs the Number and Size of Secreted Extracellular Vesicles

**Fig. 2:**
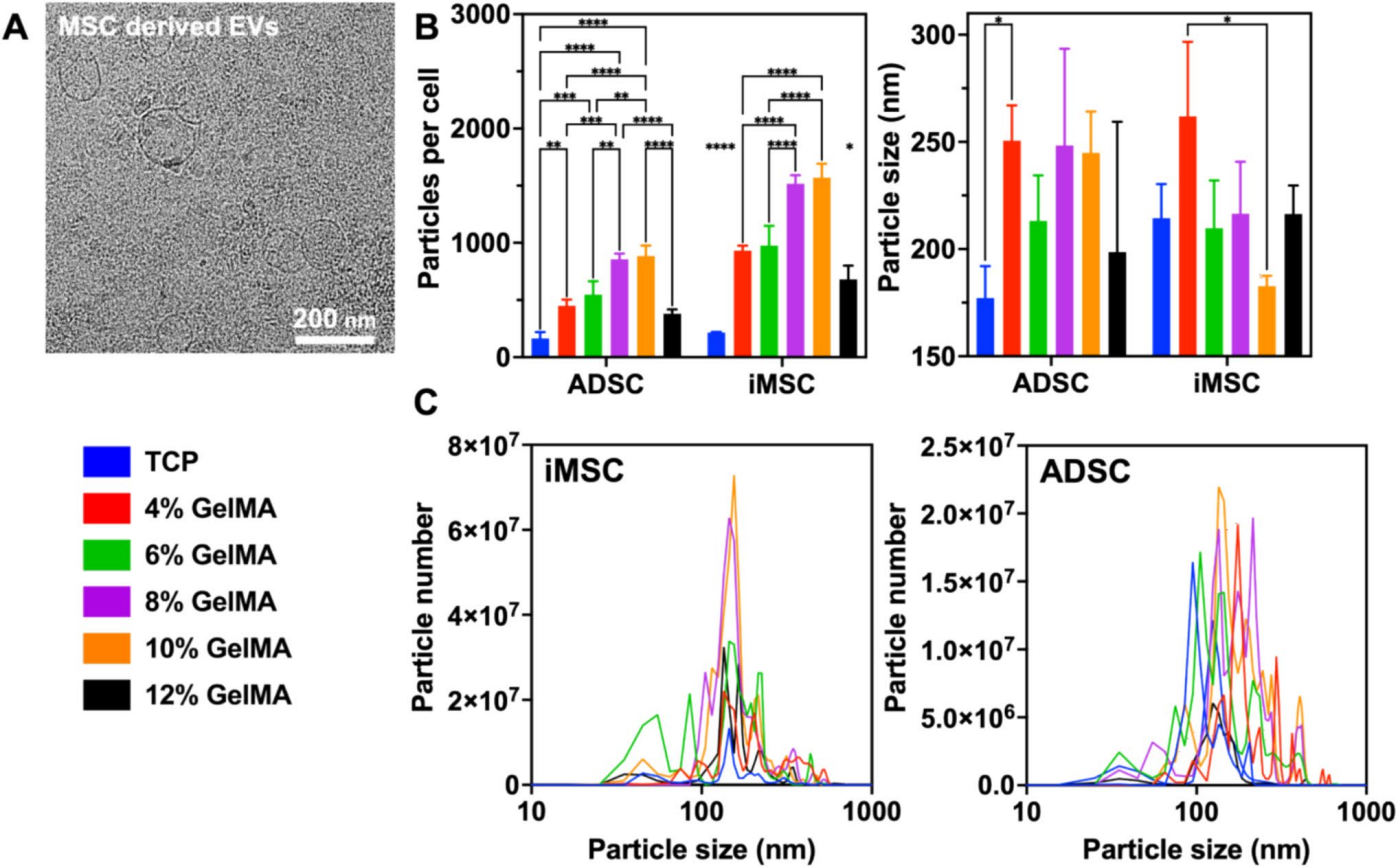
Hydrogel stiffness influences extracellular vesicle secretion. **A**. Cryo-Transmission electron microscopy (Cryo-TEM) image of EVs secreted from iMSCs, Scale bar = 200 µm. **B**. EV number per cell and size vary as a function of cell type and substrate stiffness. Statistical significance determined with *p < 0 .05, **p < 0 .01, ***p < 0.001and ****p < 0.0001. **C**. Nanoparticle tracking analysis measurements of EV particle size

To evaluate EV secretion, ADSCs and iMSCs were cultured on coverslips coated with 2D GelMA hydrogels for 24 hours, allowing cell adhesion and spreading. We chose to evaluate 2D cell culture as a first step to compare to conventional tissue culture plates and to approximate the interface a cell would experience at a microcarrier. After the initial culture period, the medium was replaced with LG DMEM to induce the production of MSC-conditioned media (MSC-CM). The MSC-CM was then centrifuged and filtered through a 0.45 µm membrane to eliminate cell debris and larger particles. EVs were subsequently isolated and analyzed using cryo-transmission electron microscopy (cryo-TEM), which confirmed their size to be under 200 nm with a typical spherical shape (Figure 2A).

Quantification was performed using nanoparticle tracking analysis (Nanosight NS300), revealing that 10% GelMA hydrogels corresponding to a stiffness of approximately 10-12 KPa produced the highest EV yield. Specifically, ADSCs secreted approximately 5.4-fold more EVs, while iMSCs produced around 7.3-fold more EVs, when compared to other GelMA concentrations (Figure 2B). These results suggest that there is an optimal mechanical microenvironment for augmenting EV secretion, irrespective of MSC origin. Average EV size and median diameter remained below 300 nm across all conditions, with no discernible trend relative to GelMA concentration or cell type (Figure 2B–C, Figure S1A). The findings highlight the critical role of mechanical cues in regulating secretory activity in the cellular microenvironment, which can be mimicked to increase EV output.

### Packed Suspensions of Hydrogel Microcarriers Influence Cell Adhesion and Morphology

While 2D platforms are effective for small-scale experiments, they pose limitations with respect to scale up for clinical or industrial-scale cell and EV production. To overcome these challenges, researchers are increasingly turning to 3D culture platforms, which offer a more scalable and efficient alternative to monolayer cultures. Not only do 3D systems reduce spatial and operational constraints, but they also enhance EV secretion and potency, likely due to improved cell-cell and cell-matrix interactions that better mimic the native tissue environment [33–35].

**Fig. 3:**
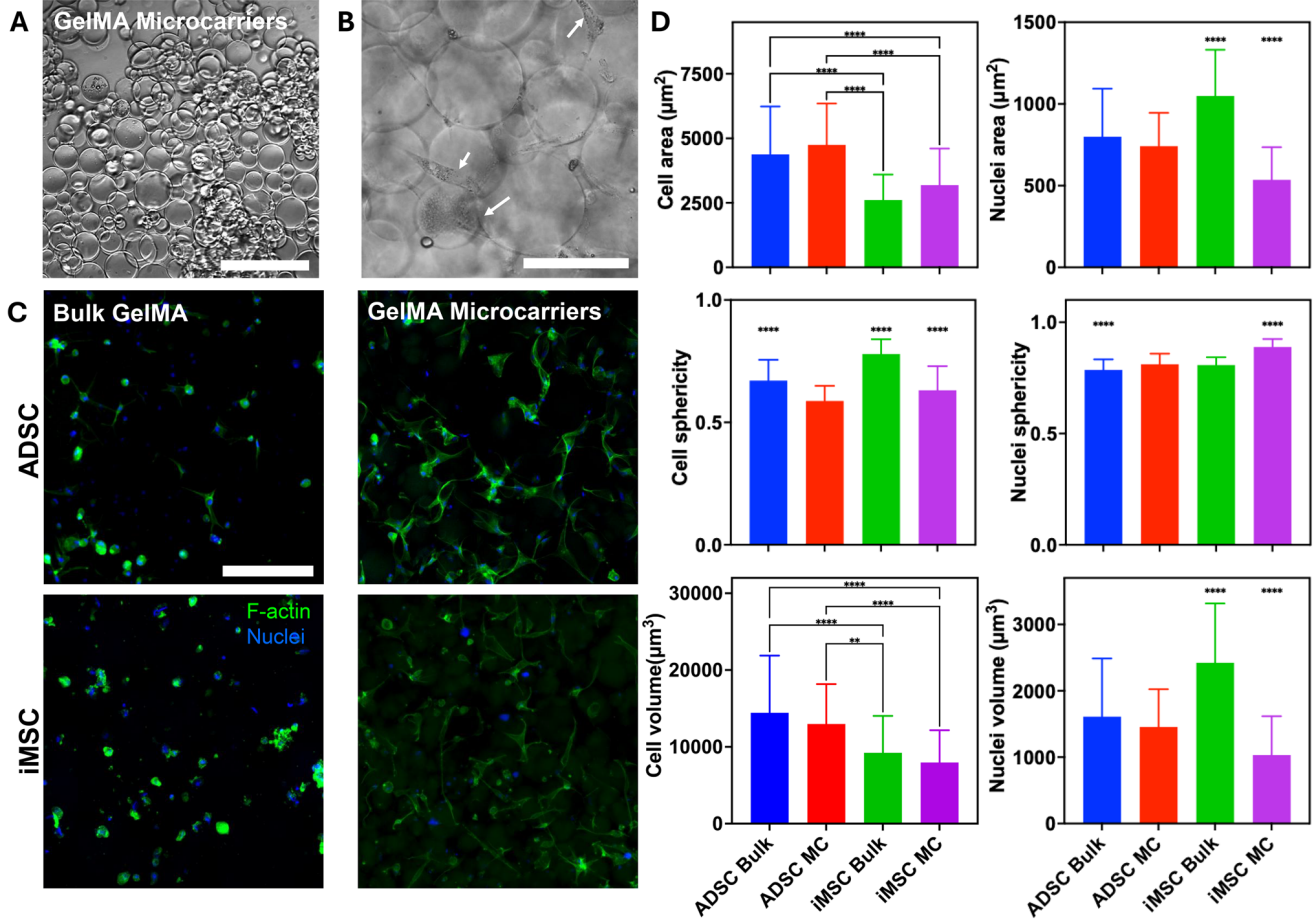
Hydrogel micro-structuring increases cell adhesion with changes in cell and nuclear morphometrics. **A**. Schematic of changes in cell morphology in GelMA bulk versus GelMA microcarriers, Scale bar = 100 µm. **B**. Brightfield microscopy image of GelMA microcarriers, Scale bar = 50 µm. **C**. Immunofluorescence images of ADSCs and iMSCs in GelMA bulk and GelMA microcarrier hydrogel suspensions Scale bar = 200 µm. **D**. Quantification of cell and nuclear morphometrics in bulk and microcarrier GelMA hydrogels. Statistical significance determined with *p < 0 .05, **p < 0 .01, ***p < 0.001and ****p < 0.0001

Selection of a 3D cell culture matrix is driven by conditions that foster favorable growth and desirable cellular activities. Encapsulation of cells in hydrogels can limit cell spreading, an important criterion for EV production, and can entrap the released EVs. Alternatively, microcarriers have proved a versatile 3D culture substratum in bioreactors to ensure a high density of cells and ease of EV harvest. GelMA microcarriers were synthesized using a water-in-oil emulsion method to overcome the limitations of bulk GelMA hydrogels in cell culture (Fig. 3A). ADSCs and iMSCs were encapsulated in 10% GelMA microcarriers and UV-crosslinked to form packed microgel suspensions. These suspensions offer a 3D microenvironment that supports MSC viability, provides macro-porosity for efficient nutrient and gas exchange, and allows space for cell spreading (Fig. 3B). This microcarrier-based approach enhances cell-matrix interactions and is better suited for scalable EV production and harvest.

After one day of cell culture followed by one day of conditioned media collection, MSCs cultured in GelMA bulk hydrogels and GelMA microcarriers were analyzed for morphometric changes (Fig. 3C). Cells adherent to microcarriers exhibited reduced nuclear area and volume, alongside a modest increase in nuclear sphericity, regardless of cell line. (Fig. 3D). Additionally, overall cell area increased, while cell sphericity and volume decreased. These observations highlight the inherent plasticity of MSCs and their sensitivity to the dimensionality of the substrate, emphasizing how 3D microenvironments can influence cellular morphology with potential impacts on extracellular vesicle (EV) secretion profiles.

### Microcarrier Culture Leads to Increased Secretion of Extracellular Vesicles with Broader Size Distributions

**Fig. 4:**
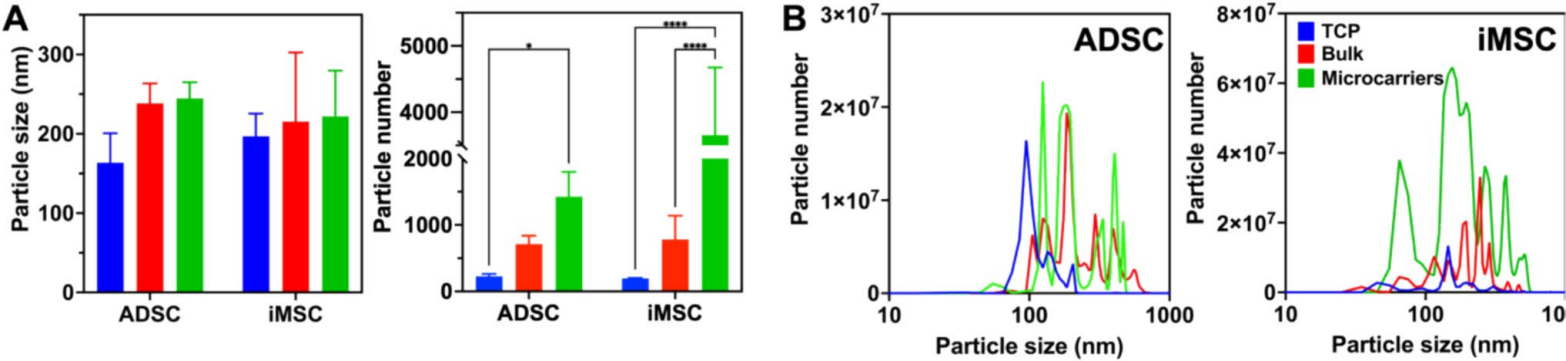
Cell adhesion in microcarrier culture influences the size and number of secreted EVs. **A**. Particle size and EV number per cell for each cell source. **B**. Nanoparticle tracking measurements of EV particle size for ADSCs and iMSCs. Statistical significance determined with *p < 0 .05, **p < 0 .01, ***p < 0.001 and ****p < 0.0001

To assess EV secretion, MSCs were cultured for 24 hours in MSC expansion media using both bulk and microcarrier GelMA hydrogels, allowing for cell adhesion and spreading. MSC-CM was then collected and analyzed for EV output. Compared to tissue culture plastic (TCP), ADSCs secreted approximately 3.3-fold more EVs in bulk GelMA and 6.8-fold more in microcarrier conditions. iMSCs showed even greater enhancement, releasing approximately 7.6-fold more EVs in bulk and 17.7-fold more in microcarrier cultures (Fig. 4A). The substantial increase in EV output observed in microcarrier cultures may be attributed to the improved cell-matrix interactions and enhanced nutrient diffusion afforded by the 3D microenvironment. In contrast, the lower EV recovery from bulk hydrogels could be due to physical and biochemical constraints, including limited pore size and barriers to diffusion, which may trap EVs within the hydrogel matrix. Additionally, EV-matrix interactions such as physical adsorption or specific matrix binding may further hinder vesicle release.

Interestingly, we observed a marked increase in particles < 300 nm from microcarrier cultures (Fig. 4A–B, Fig. S1B). This trend highlights the influence of substrate dimensionality beyond stiffness alone on EV biogenesis and size distribution. The enhanced EV secretion and prevalence of smaller vesicles from culture in microcarrier systems aligns more closely with natural tissue secretory profiles, which may be attributed to improved physiologically mimicry provided by the 3D microenvironment. These cues include substrate stiffness within the soft-tissue physiological range (~0.1-15 KPa, compared to the ~ GPa stiffness of planar polystyrene) [24], 3D matrix anchorage [36], and surface curvature [27], all of which influence cytoskeletal tension and membrane dynamics. Additionally, features such as matrix viscoelasticity [37, 38], which modulates cell spreading and contractility, and microcarrier porosity and degradability [39], can further regulate endosomal trafficking and vesicle budding pathways. Collectively, these mechanical and structural attributes of microcarriers more closely mimic native tissue environments and may contribute to the observed bias toward smaller EV populations.

These findings underscore the potential of microcarrier-based platforms not only to boost EV yield but also to support scalable and clinically relevant EV production. By mimicking native tissue architecture and enabling efficient nutrient and oxygen exchange, 3D GelMA microcarriers offer a promising strategy for advancing EV-based therapeutics in regenerative medicine and beyond.

### Microcarrier Culture Influences Protein Content in Secreted Extracellular Vesicles

To characterize the protein cargo of EVs derived from ADSCs and iMSCs, a proteomic analysis was performed using LC-MS/MS [1]. EVs were isolated from conditioned media collected after culturing cells on TCP and GelMA microcarriers. Remarkably, nearly > 90% of the proteins identified in EVs from both cell types matched entries in the ExoCarta database, confirming the reliability and specificity of the isolation. A total of 117 proteins were found to be common across all conditions, while distinct protein signatures emerged depending on the substrate. Specifically, 18 and 22 unique proteins were identified in ADSC-EVs from TCP and microcarriers, respectively, and 26 and 25 unique proteins were found in iMSC-EVs from the same conditions. These unique protein profiles suggest that substrate dimensionality influences EV cargo, potentially altering their biological function and therapeutic potential.

**Fig. 5:**
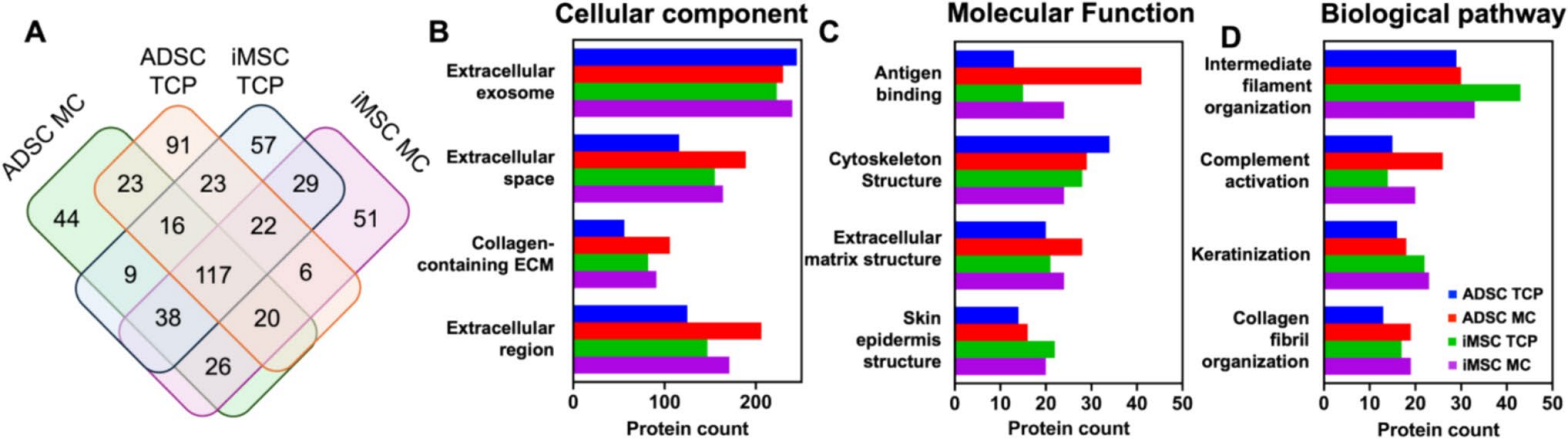
Proteomics analysis of cell secretome when cultured on tissue culture plastic or in 3D microcarrier culture. **A**. Venn diagram analysis of differential protein expression across conditions. B-D Detected protein analysis as a function of cellular component (**B**), molecular function (**C**) and biological pathway (**D**)

The gene ontology (GO) analysis grouped the results as per cellular component, molecular function and biological pathway. In the cellular component category, proteins were significantly enriched in extracellular exosome, extracellular space, and extracellular region, confirming the vesicular nature of the isolates (Fig. 5B). In terms of molecular function, EVs derived from microcarriers showed notable enrichment in antigen binding and extracellular matrix (ECM) structural components, regardless of cell source. Meanwhile, iMSC-EVs irrespective of substrate were enriched in proteins associated with skin epidermis structure (Fig. 5C), suggesting a potential utility in epithelial regeneration. Biological pathway analysis revealed that EVs from microcarriers were predominantly involved in complement activation and collagen fibril organization, both critical for wound healing and tissue remodelling. Additionally, EVs from iMSCs across substrates were enriched in pathways related to skin keratinization (Fig. 5D). The prominence of epidermal and skin-related proteins in iMSC-EVs aligns with the dermal fibroblast origin of the iPSC lines used to generate the iMSCs and may also reflect residual epigenetic memory retained from the parental fibroblasts [40].

These findings collectively indicate that MSC-EVs, particularly those produced in 3D microcarrier environments, are functionally equipped to support wound healing and tissue regeneration processes. The substrate-dependent enrichment of specific protein cargos highlights the importance of culture conditions in tailoring EVs for targeted therapeutic applications.

### Functional Tests Indicate Increased Activity in Extracellular Vesicles from Microcarrier Culture

**Fig. 6:**
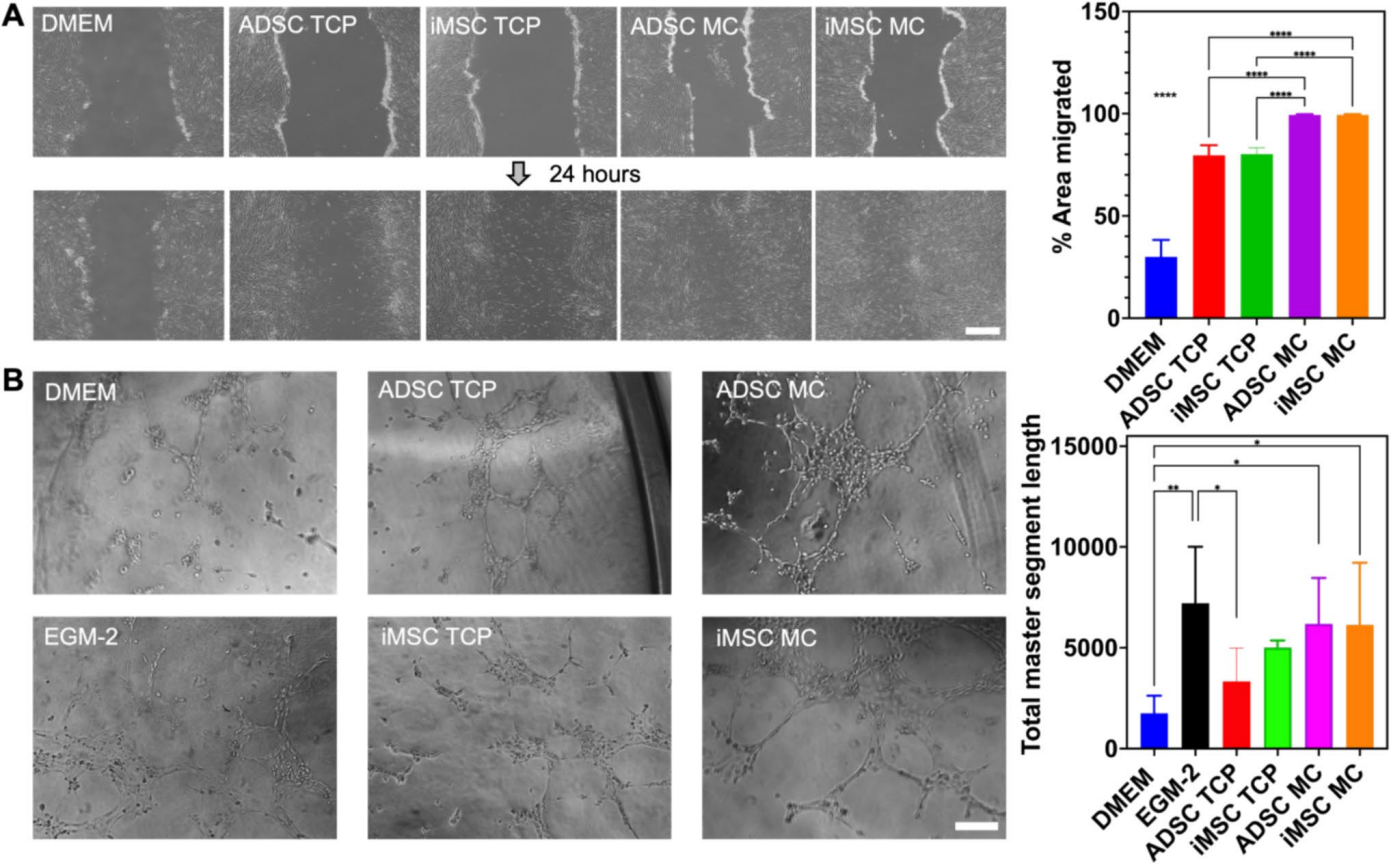
Functional assessment of EV activity in models of wound healing and angiogenesis. **A**. Photographs of scratch assays and quantitation for ADSCs and iMSCs cultured on tissue culture plastic or in GelMA microcarrier culture, Scale bar = 200 µm. **B**. Photographs of endothelial tubulogenic assays and quantitation for ADSCs and iMSCs cultured on tissue culture plastic or in GelMA microcarrier culture Scale bar = 100 µm. Statistical significance determined with *p < 0 .05, **p < 0 .01, ***p < 0.001 and ****p < 0.0001

Given that proteomic analysis revealed enrichment of proteins associated with wound healing and tissue remodelling, we proceeded to evaluate the functional potency of the EVs through two well-established in vitro assays. First, a fibroblast scratch assay was conducted to assess the EVs’ ability to promote cell migration and wound closure, key indicators of regenerative potential. Upon reaching monolayer confluency, multiple linear scratches were introduced using a 200 μL pipette tip to simulate wound gaps. EVs derived from various cell sources and substrate conditions including TCP and GelMA microcarriers were added to the cultures. After a 24-hour incubation period, wound closure was quantified, (Fig. 6A). EVs from microcarrier cultures demonstrated markedly superior healing efficacy, achieving ~ 99% closure of the scratch area corresponding to higher EV number. In contrast, EVs from TCP conditions resulted in ~ 80% closure. These results highlight the enhanced bioactivity of EVs produced in 3D microcarrier environments, consistent with increased secretion of EVs, with enriched cargo. We should note that while the number of seeded cells was maintained across conditions, differences in proliferation and EV secretion per cell will likely change. To quantitatively assess cargo activity, future studies should normalize EV numbers prior to functional tests.

To further assess regenerative potential, endothelial tube formation assays were conducted. Human microvascular endothelial cells (HMVECs) were seeded on Geltrex-coated plates and treated with EVs. After 8 hours, microcarrier-derived EVs promoted significantly greater tube formation than TCP-derived EVs, indicating enhanced angiogenic activity (Fig. 6B). These results highlight the enhanced bioactivity of EVs produced in 3D microcarrier culture, likely due to elevated EV secretion, from optimized microenvironments.

## Conclusion

Mesenchymal stem cell derived extracellular vesicles (EVs) are a promising therapeutic avenue for a broad range of conditions ranging from acute and chronic wound healing, graft versus host disease, musculoskeletal, cardio vasculature and neurological disorders, immune diseases, and degenerative conditions. Here we used gelatin methacrylate (GelMA) as a cell culture material and showed how dimensionality, stiffness and composition influence EV yield, size characteristics and molecular content. Cell source played a role in defining the molecular fingerprint of the EVs and should be considered when optimizing conditions for specific therapeutic applications. Importantly, we found that soft gelatin-based microcarriers could be tuned to maximize EV secretion up to 18-fold compared to monolayer cultures. This dramatic increase points to the promise in using hydrogel micro carriers in production and manufacturing processes. GelMA is one of the most commonly used hydrogels with GMP grade materials readily available. Hydrogel-based microcarrier culture systems are poised to have a major impact in the production of therapeutic cells and cell-based products in advanced manufacturing.

## Supplementary Information

Below is the link to the electronic supplementary material.

Supplementary file1 (DOCX 83 KB)

## Data Availability

The datasets used and analyzed during the current study are available from the corresponding author on reasonable request.
