# Supplementary material for: Tuning Hydrogel Mechanics and Microstructure to Maximize Extracellular Vesicle Production from Mesenchymal Stem Cells
Source: Cell Mol Bioeng. 2026 Jun 3;19(3):375–86. doi: 10.1007/s12195-026-00917-x (PMC13365067; doi:10.1007/s12195-026-00917-x)
Supplement: Supplementary file 1 — Supplementary file1 (DOCX 83 KB) [file 12195_2026_917_MOESM1_ESM.docx]

**Supplementary data:**

| GelMA hydrogels vs TCP | 4% | 6% | 8% | 10% | 12% |
| --- | --- | --- | --- | --- | --- |
| ADSC | 2.7 | 3.3 | 5.2 | 5.4 | 2.3 |
| iMSC | 4.4 | 4.5 | 7.2 | 7.3 | 3.1 |

Table 1 Comparison of EV secreted by ADSCs and iMSCs when cultured on 2D GelMA hydrogels vs TCP respectively

| EVs secreted across cell lines | TCP | 4% | 6% | 8% | 10% | 12% |
| --- | --- | --- | --- | --- | --- | --- |
| iMSC vs ADSC | 1.3 | 1.4 | 1.8 | 1.8 | 1.8 | 1.8 |

Table 2 Comparison of EVs secreted between ADSCs and iMSCs across different 2D GelMA hydrogels and TCP respectively

| GelMA hydrogels vs TCP | Bulk | Microcarriers |
| --- | --- | --- |
| ADSC | 3.3 | 6.8 |
| iMSC | 7.6 | 17.7 |

Table 3 Comparison of EV secreted by ADSCs and iMSCs when cultured on different 3D GelMA hydrogels vs TCP respectively

| EVs secreted across cell lines | TCP | Bulk | Microcarriers |
| --- | --- | --- | --- |
| iMSC vs ADSC | 1.2 | 2.3 | 2.6 |

Table 4 Comparison of EVs secreted between ADSCs and iMSCs across different 3D GelMA hydrogels and TCP respectively


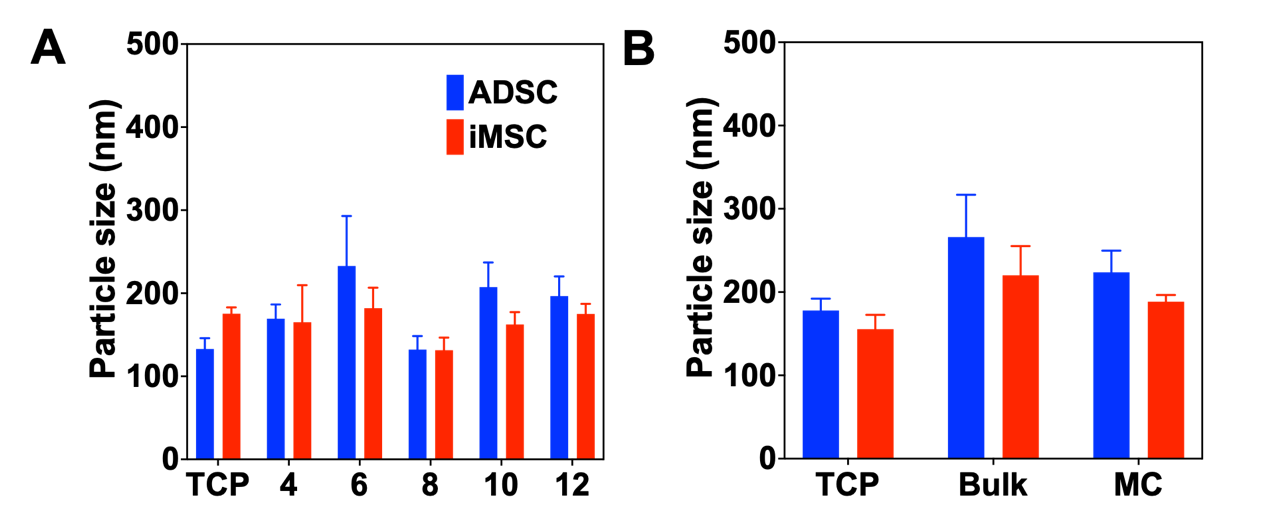


**Figure S1**. Median particle diameter across each cell source using nanoparticle tracking analysis A. 2D GelMA hydrogels and B. Microcarrier culture. Statistical significance determined with **p* <0 .05, ***p* <0 .01, ****p* < 0.001and *****p* < 0.0001
